# Bacteriological Profile and Antimicrobial Resistance Patterns in Neonatal Sepsis: Special Reference to Klebsiella pneumoniae at a Tertiary Care Hospital in Eastern India

**DOI:** 10.7759/cureus.91735

**Published:** 2025-09-06

**Authors:** Biyanka Sau, Srijita Ghosh, Subhranil Mal, Paramita Adhikary

**Affiliations:** 1 Microbiology, Medical College Kolkata, Kolkata, IND

**Keywords:** antibiotic stewardship, antimicrobial susceptibility, klebsiella pneumoniae, multidrug resistance, neonatal intensive care unit, neonatal sepsis

## Abstract

Background

Neonatal sepsis remains a significant cause of morbidity and mortality among infants ≤ 28 days of age, especially in developing and underdeveloped countries, ranking as the third major cause of death among neonates. *Klebsiella pneumoniae* is a Gram-negative bacterium of the family Enterobacteriaceae, a frequent pathogen in sepsis, and often exhibits multi-drug resistance (MDR), thereby complicating therapeutic options and prolonging hospital stay. This study aimed to evaluate the current trends in the bacteriological profile of neonatal sepsis and assess antimicrobial susceptibility patterns, with special emphasis on *K. pneumoniae* and its MDR status in a tertiary care NICU setting.

Methods

This prospective cross-sectional study was conducted over six months in the Neonatal Intensive Care Unit (NICU), Medical College, Kolkata. Blood cultures were obtained from 100 neonates with clinically suspected sepsis and processed using the automated system for microbial identification and antibiotic susceptibility testing. Data analysis was performed using Microsoft Excel (Microsoft® Corp., Redmond, WA) and SPSS Statistical Product and Service Solutions (SPSS, version 20.0; IBM SPSS Statistics for Windows, Armonk, NY).

Results

Culture positivity was observed in 66% of the cases. *K. pneumoniae* was the most predominant organism with 16% of all cases; 93.75% of these isolates were identified as multidrug-resistant. High resistance rates were observed against a wide array of commonly used antibiotics such as cephalosporins, aminoglycosides, carbapenems, and aztreonam. Other notable pathogens isolated were *Acinetobacter baumannii* (15%) and *Burkholderia cepacia* (8%), both showing considerable resistance patterns.

Conclusion

This study reveals the growing burden of multidrug-resistant *K. pneumoniae* in neonatal sepsis and highlights the immediate need for antibiotic stewardship and infection control practices. Early and accurate identification of causative organisms and their resistance profiles is essential in guiding treatment modalities and curbing the spread of infections in neonatal care settings.

## Introduction

Neonatal sepsis defines systemic infection occurring in infants ≤ 28 days of age. It is typically caused by bacterial, viral, fungal, or parasitic organisms, resulting in several hemodynamic changes and clinical symptoms, which cause severe morbidity and mortality [1]. With incidence ranging from one to 20 per 1,000 live births and a high mortality rate of about 11-19%, the clinical presentation is influenced by a number of factors, including the type and virulence of the pathogen, the portal of entry, the host’s immune response, and the progression of the underlying health condition [2]. Typical signs and symptoms include fever, vomiting, diarrhea, irritability, lethargy, respiratory distress, hypoglycemia, jaundice, poor feeding, and seizures [3].

Neonatal sepsis may be classified according to the time of onset of the disease: early onset and late onset. Early-onset neonatal sepsis (EONS) is defined as bacteraemia occurring within 72 hours in pre-term infants or seven days in term infants, whereas late-onset sepsis (LONS) develops beyond 72 hours in preterm infants and after seven days of life in term infants [1]. The spectrum of causative pathogens in neonatal sepsis is influenced by both geographic variation and temporal trends. Though early initiation of antimicrobial therapy is crucial, its success is increasingly limited by rising antimicrobial resistance. Consequently, the timely identification of infection, through the use of reliable diagnostic biomarkers, is imperative for guiding appropriate therapeutic interventions in both early-onset and late-onset sepsis.

This prospective cross-sectional study aimed to determine the causative pathogens responsible for neonatal septicemia and evaluate their patterns of antibiotic susceptibility. The difficulties in diagnosing sepsis often result in erratic and irrational use of antibiotics, contributing to the emergence of multidrug-resistant (MDR) organisms. A significant percentage of deaths are due to MDR pathogens, thus complicating clinical management of sepsis. Hence, understanding the risk factors, clinical features, microbial etiology, and their antibiotic sensitivity pattern becomes crucial to guide management and promote antibiotic stewardship [4]. This study reports the findings based on comprehensive clinical assessments, microbiological analysis of sepsis, and antimicrobial susceptibility testing done in septicaemic neonates admitted to a tertiary care hospital.

## Materials and methods

Study design and setting

Ethical approval for the study was obtained from the Institutional Ethics Committee (IEC) of Medical College, Kolkata (Memo Number: MC/KOL/IEC/NON-SPON/2554/07/2024). The study was conducted in the Neonatal Intensive Care Unit (NICU) and Department of Microbiology, Medical College, Kolkata, India, for a period of six months - from June 2024 to December 2024.

Study population

A total of 100 neonates with sepsis were included in the study. The diagnosis was made based on clinical features and laboratory findings in accordance with the standard infant sepsis criteria.

Inclusion and exclusion criteria

Neonates aged ≤28 days who exhibited clinical signs of sepsis, such as lethargy, temperature instability, respiratory distress, poor feeding, or irritability were included in the study, while those with congenital anomalies or already receiving antibiotic therapy were excluded from participation.

Sample collection and laboratory analysis

Blood samples were collected from 100 neonates suspected of developing sepsis, shortly after their admission to the NICU. These samples were collected into automated pediatric blood culture bottles and incubated in the BacT/Alert 3D system. Bottles that flagged positive were further processed for microbial identification and antibiotic susceptibility testing using the VITEK 2 compact system (bioMérieux, Marcy-l’Étoile, France). For example, *Klebsiella *spp. isolates were identified and antibiotic sensitivity was tested using the VITEK 2 cards (VITEK2 GN ID, VITEK2 AST-N406 and VITEK2 AST-N407), following the interpretive criteria standardized for minimum inhibitory concentration (MIC) of antibiotics using the Clinical and Laboratory Standards Institute (CLSI) and the European Committee on Antimicrobial Susceptibility Testing (EUCAST), as integrated within the VITEK 2 software (bioMérieux, Marcy-l’Étoile, France).

Statistical analysis

The study-specific data were collected in respective case study forms (CSF). The data from the CSF were then transcribed onto a Microsoft Excel database (Microsoft® Corp., Redmond, WA), and statistical analysis was done in Statistical Product and Service Solutions (SPSS, version 20; IBM SPSS Statistics for Windows, Armonk, NY).

## Results

**Figure 1:**
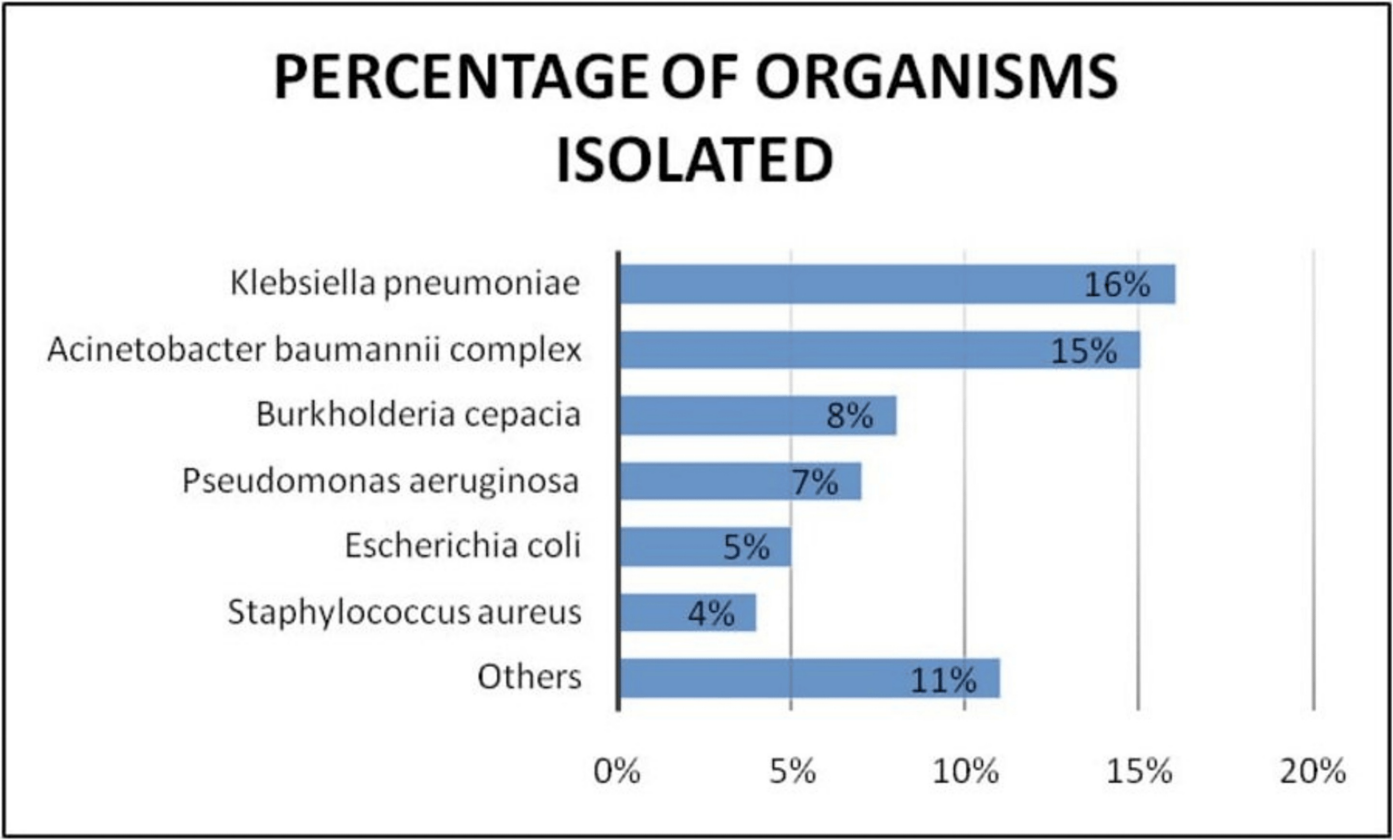
Bar graph showing the distribution of bacterial isolates in cases of neonatal sepsis under study

Microbiological profiles from different studies on neonatal sepsis show a wide range of organisms, both Gram-positive and Gram-negative, as the underlying causative agents. The findings in the present study also reveal a similar picture, with *K. pneumoniae* being the most commonly isolated organism, contributing to 16% of all the cases, as shown in Figure 1. This was closely followed by *Acinetobacter baumannii* complex (15%), *Burkholderia cepacia* (8%), *Pseudomonas aeruginosa* (7%), *Escherichia coli* (5%), and *Staphylococcus aureus *(4%). Notably, 34% of the cases were culture-negative. Minor organisms, such as *Cupriavidus pauculus*, *Pseudomonas stutzeri*, *Elizabethkingia meningoseptica*, and *Ralstonia insidiosa*, were also isolated, albeit in a small number of cases. Their presence may suggest potential environmental contamination or infection in immunocompromised neonates.

**Figure 2:**
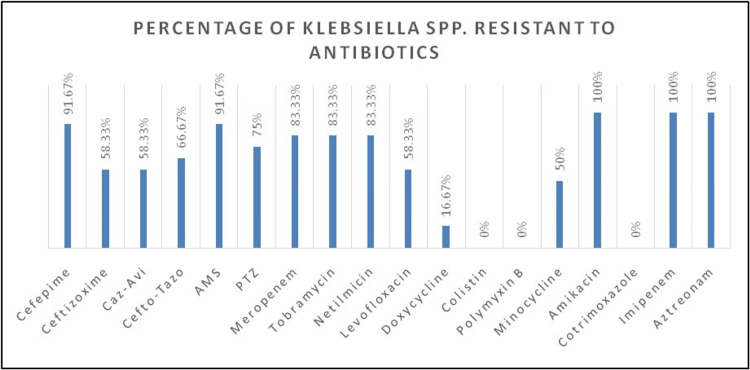
Bar graph showing antibiotic resistance patterns of Klebsiella pneumoniae Caz-Avi = Ceftazidime-Avibactam, Cefto-Tazo = Ceftolozane-Tazobactam, AMS = Ampicillin-Sulbactam, PTZ = Piperacillin-Tazobactam

The predominance of resistant *Klebsiella* spp. in the culture isolates was particularly concerning, as it may be suggestive of suboptimal sterilization practices and lapses in antibiotic stewardship. In the current study, *Klebsiella* isolates demonstrated notably high resistance to a wide array of antibiotics, including cephalosporins, beta-lactam/beta-lactam inhibitor combinations, fluoroquinolones, carbapenems, and aminoglycosides, as demonstrated in Figure 2. Significant resistance was seen against amikacin (100%), imipenem (100%), aztreonam (100%), cefepime (91.67%), ampicillin-sulbactam (91.67%), meropenem (83.33%), tobramycin (83.33%), netilmicin (83.33%), piperacillin-tazobactam (75%), and several others. Intermediate sensitivity was noted in some isolates against doxycycline, levofloxacin, minocycline, meropenem, colistin, and polymyxin B, while sensitivity was observed only against a small subset of antibiotics - most notable being cotrimoxazole.

A closer inspection into the resistance profile of the *Klebsiella* isolates reveals the presence of MDR organisms - bacteria that have acquired resistance to at least one agent in three or more classes of antimicrobial agents [5]. Among the 16 *Klebsiella* isolates, a staggering 93.75% exhibited MDR patterns, with one isolate even being pan-resistant to all tested antibiotics except doxycycline. Such resistance arises due to the production of inactivating enzymes, mutation of target sites, development of efflux pumps, reduced membrane permeability, and other adaptive mechanisms. The presence of these organisms complicates pharmacotherapy, limiting antibiotic options and facilitating rapid spread among susceptible individuals.

Among other pathogens, *A. baumannii* was found to be significantly resistant to carbapenems (93.3%), third- and fourth-generation cephalosporins (86.7%), fluoroquinolones (80%), and aminoglycosides (73.3%). The eight isolates of *B. cepacia* showed notable resistance to ceftazidime, cefepime, and meropenem, with 75% of them qualifying as MDR. *E. coli* isolates demonstrated sensitivity to carbapenems (80%), beta-lactam/beta-lactamase inhibitor combinations (60%), and amikacin (80%) but were resistant to the other antibiotics tested.

## Discussion

Neonatal sepsis remains a significant concern in NICUs due to its high rates of morbidity and mortality. Characterized by complications such as septicaemia, meningitis, pneumonia, arthritis, osteomyelitis, and urinary tract infections, it remains a global health concern among researchers and medical professionals, largely due to its high incidence and alarming infectivity and spread. Attia Hussein Mahmoud et al. [6] reported that neonatal sepsis is the third major cause of neonatal deaths, culminating in 203,000 deaths per year. A systematic review and meta-analysis (SRMA) done by Li et al. [7] further revealed a 12.79% increase in the global burden of neonatal septicaemia and related infections from 1990 to 2019 [7]. This was particularly demonstrable in middle- and low-income countries, as reported by Dessu et al. [8] and Mukherjee et al. [9], with neonatal sepsis accounting for 30-35% of neonatal deaths, next to prematurity and its complications.

A clear understanding of the microbial profile is, therefore, essential for guiding effective clinical management. The causative organisms are manifold, with various organisms contributing to the disease burden. Saha et al. [1] in a research article noted that, among suspected cases of septicaemia, most yielded growth of organisms such as *Candida* spp., *Klebsiella* spp., *E. coli*, *S. aureus*, *Enterococcus* spp., and others [1]. Another study conducted in Kolkata by Chakraborty et al. [10] showed that the most common isolates in cases of neonatal sepsis were organisms of the Enterobacterales group and non-fermenters. *E. coli *identified were found to be resistant to second-generation cephalosporins, beta-lactams, fluoroquinolones, and cotrimoxazole, while *Enterococcus* spp. from ICUs were especially resistant to ampicillin and fluoroquinolones [10].

The National Neonatal-Perinatal Database (NNPD) report, encompassing data from 151,436 deliveries across 18 centres over two years, reveals that, among systemic infections, septicemia was the most common clinical presentation (88.1%), with pneumonia diagnosed in 32.8% of sepsis cases. *K. pneumoniae* was the most frequently isolated organism out of the 1,248 culture-positive infants, leading to 32.5% of all the cases - followed by *S. aureus* (13.6%) and *E. coli *(10.6%). Alarmingly, most strains of* Klebsiella* showed poor sensitivity to antibiotics, including amikacin (31.23%), ceftazidime (7.9%), ciprofloxacin (26.8%), cefotaxime (24.0%), and gentamicin (22.6%), emphasizing the growing challenge of antimicrobial resistance in neonatal care [11]. Zelellw et al. [12] also described *Klebsiella* spp. as the leading pathogen behind neonatal sepsis in developing countries. Similar findings were echoed in the review of the etiology of community-acquired neonatal sepsis in low- and middle-income countries by Waters et al. [13].

As disclosed by the aforementioned studies and SRMAs, *K. pneumoniae* is thus a major causative agent in the context of neonatal sepsis, causing both community and healthcare-associated infections [6-13]. It is a rod-shaped Gram-negative, non-motile, lactose-fermenting, facultative anaerobic bacterium. *Klebsiella*, a documented cause of LONS, is known to produce massive outbreaks in NICUs and hospital setups. This is attributable in part to their plasmid-mediated MDR, extended-spectrum beta-lactamase (ESBL) production, and biofilm formation, adding to their persistence and transmission in hospital environments.

Mukherjee et al. [9] illuminated upon this virulent nature of *Klebsiella* spp., highlighting the growing threat of carbapenem-resistant *K. pneumoniae* (CRKP) and hypervirulent *K. pneumoniae* (hvKP) in neonatal sepsis - especially in low- and middle-income countries (LMICs). With the development of resistance against other antibiotics, the increasing dependency on carbapenems has led to the emergence of CRKP, mediated by highly transmissible plasmid-encoded carbapenemase enzymes, including KPC, NDM, and OXA-48-like enzymes. A more invasive variant, hvKP, poses additional questions for physicians, while the convergence of CRKP and hvKP further amplifies this problem [9]. In another cohort study conducted in Cairo, *K. pneumoniae* turned out to be the most common pathogen in LONS cases, and 56.5% of these isolates were resistant to meropenem. OXA-48 was the most prevalent carbapenemase enzyme, accounting for 60.8% cases, while NDM-1 was found in 52.2% cases as detected by multiplex PCR - with more than half of the isolates co-harbouring both [14]. The current study also documents a high level of resistance to carbapenems, one of the "last-resort antibiotics" - 83.3% against meropenem and total insensitivity to imipenem.

Development of such resistance leads to difficulty in treating infections, resulting in prolonged illness and hospitalisation, increased mortality and higher healthcare costs. The Antibiotic Resistance and Prescribing in European Children (ARPEC) study, conducted at 226 hospitals in 41 countries, reported that 40% of the pathogens isolated were resistant to first-line antibiotics prescribed by WHO, with MDR organisms frequenting between 30% and 50% [15]. In another article, Yusef et al. [16] observed multidrug resistance in 69% isolates, with such organisms leading to significantly higher mortality (60%) compared to drug-sensitive bacteria [16]. High rates of multidrug resistance have also been reported in the Delhi Neonatal Infection Study (DeNIS) [17]. In line with these observations, our findings align closely with existing literature, with *Klebsiella *and *Burkholderia* isolates showing high multidrug resistance rates - 93.75% and 75%, respectively - further emphasizing the urgent need for targeted stewardship and infection control strategies.

## Conclusions

The present study underscores the emerging trends in bacteriological profile and antimicrobial resistance patterns among neonates with sepsis, highlighting a growing prevalence of MDR organisms. These findings reinforce the need for implementing effective antibiotic stewardship programs and robust infection control measures to counter escalating resistance against common antimicrobials. A comprehensive approach, thus, is of paramount importance in disentangling the ever-increasing web of resistance through rational usage of antibiotics, surveillance systems, regulation of over-the-counter (OTC) drug sales, raising public awareness, and revitalisation of the already thinned-out pharmaceutical pipeline of antimicrobial drugs. Coordination in these domains worldwide is essential to safeguard neonatal health and preserve the efficacy of existing antimicrobial therapies.
